# Alterations in articular cartilage frictional properties in the setting of acute gouty arthritis

**DOI:** 10.1371/journal.pone.0298722

**Published:** 2024-03-21

**Authors:** Pai Zheng, Xueer Zhang, Chengcheng Feng, Yuhong Yu, Guangwei Che, Zhihong Cao, Li Tian, Yong Huang

**Affiliations:** 1 Chengdu University of Traditional Chinese Medicine, Chengdu, China; 2 Hospital of Chengdu University of Traditional Chinese Medicine, Chengdu, China; 3 Key Laboratory of Testing Technology for Manufacturing Process in Ministry of Education, Southwest University of Science and Technology, Mianyang, China; University of South Carolina, UNITED STATES

## Abstract

The tribological behaviour of articular cartilage plays a key role in joint motion; however, there is a gap in research on the effect of hyperuricemic joint fluid on cartilage friction behaviour in acute gouty arthritis. In this study, we carried out a fixed-load scratch experiment to compare the friction and wear of articular cartilage under the lubrication of gouty arthritis arthritic fluid and normal human arthritic fluid, and the results showed that the cartilage friction coefficient of patients with acute gouty arthritis was significantly larger than that of normal human beings, and that the cartilage friction coefficient decreased with the elevation of normal load and sliding speed, and the change with the sliding speed varied more differently from that of normal human beings, and that the cartilage surface wear was more severe after prolonged friction. The wear and tear of the cartilage surface is more severe after prolonged friction. Patients with gouty arthritis should reduce the sudden speed changes such as fast running and variable speed running to maintain the stability of the cartilage surface friction coefficient.

## 1. Introduction

Hyperuricemia (HUA), arising from disturbances in purine metabolism, ensues when there is an excess secretion of serum uric acid (SUA) or hindered renal excretion, leading to intracorporeal uric acid accumulation and consequent HUA. Acute gouty arthritis (AGA), a commonly encountered rheumatic ailment, is intricately tied to HUA. In the course of AGA development, an abundance of monosodium urate (MSU) in synovial fluid is associated with innate immune activation [1], humoral immune dysregulation [2], oxidative stress [3], cellular apoptosis [4], and aberrations in bone metabolism [5]. This cascade, set in motion through the activation of Toll-like receptor pathways, instigates Toll-like receptors (TLRs) that trigger the TLR/MyD88/NF-κB pathway’s upstream proteins, leading to the release of downstream inflammatory factors [6]. Consequently, neutrophil-mediated inflammatory responses are incited, with direct deposition of MSU crystals on the synovial membrane’s surface. This deposition not only diminishes osteoblast vitality but also stimulates osteoclast activation, resulting in cortical breaches, culminating in osseous erosion. Clinically, this manifests as erythema, swelling, heat, and pain in the joints, accompanied by restricted mobility and cartilage degradation [7].

Articular cartilage, which blankets the surfaces of major joints such as the hip and knee, exhibits remarkable frictional reduction and minimal wear under physiological conditions owing to its biphasic solid-liquid nature. This property caters to the demands of joint mobility. The biomechanical underpinnings of osteoarticular diseases are directly linked to inadequate joint motion and internal stress distribution [8]. The incessant accumulation of wear debris from the joint’s attrition contributes to bone resorption [9]. Investigations have revealed that factors influencing cartilage lubrication encompass the cartilage surface conditions and the intrinsic components of synovial fluid [10], the velocity of cartilage friction pairs [11], and the sliding duration [12]. Building upon the biphasic solid-liquid nature of cartilage, a finite element model of cartilage with a surface amorphous layer characterized by dual phases of thin and thick layers was established [13]. This model elucidated that the thin layer effectively transfers loads from the solid phase to the liquid phase, thereby enhancing lubrication and diminishing friction. The superior mechanical properties and exceptionally low coefficient of friction (COF) of natural cartilage remain unrivaled by any existing joint prostheses or cartilage repair materials. Thus, beginning with fundamental exploration, the investigation of the biomechanical frictional properties of articular cartilage has become a focal point for scholars worldwide, aiming to safeguard the integrity of natural cartilage against deterioration.

To address this, the present study leveraged nanoindentation techniques, employing both normal synovial fluid and high MSU content synovial fluid from AGA patients as lubricants. This approach facilitated the microscopic-scale acquisition of friction coefficients between articular cartilage surfaces under various normal loads and sliding velocities. Furthermore, the influences of normal load and sliding velocity on frictional behavior were explored. The insights gained from this study contribute to the theoretical foundation for advocating active control of uric acid levels and the implementation of appropriate exercise regimens for individuals with acute gouty arthritis. This, in turn, serves to mitigate articular cartilage wear and provides empirical support for such interventions.

## 2. Materials and methods

### 2.1 Materials

To ensure material freshness and comparability, the cartilage specimens utilized in this experiment were sourced from the femoral condyles of freshly slaughtered adult pig knee joints on the same day. The intact cartilage covering the femoral condyles was carefully excised, dissociated from the joint, and cleared of surface soft tissue and oil layers. Subsequently, utilizing a friction table fixture, rectangular bone blocks measuring 5mm × 5mm × 10mm were further segmented from the intact femoral condyles to serve as pin materials. Additionally, cartilage samples measuring 30mm × 15mm × 2mm were excised as counterpart specimens for abrasive wear. These cartilage samples were preserved in a chilled PBS solution, removed half an hour prior to experimentation, and thawed at room temperature.

Given the limited availability of normal knee synovial fluid, a small residual effusion from the knee joints of early-stage chronic osteoarthritis patients, who had undergone sodium hyaluronate injection four weeks prior, was utilized as normal synovial fluid. For comparison, synovial fluid was collected from knee joints of acute gouty arthritis patients (blood SUA concentration > 420 μmol/L). Similar synovial fluids were pooled after collection, mixed, and stored under low temperatures. All experiments were conducted within 24 hours of fluid collection. Study subjects were drawn from patients at the Orthopedics Clinic of the Sichuan Provincial Hospital of Traditional Chinese Medicine from October 2021 to October 2022, and all subjects signed a written informed consent form. The study protocol was reviewed and approved by the Medical Ethics Committee of Chengdu University of Traditional Chinese Medicine Affiliated Hospital, Ethical Approval Number: 2020KL-055.

### 2.2 Nanoindentation experiment

The experiments were conducted using a nanoindenter (G200, Keysight, USA). Normal synovial fluid and synovial fluid from AGA patients were employed as lubricants. Cartilage pin specimens underwent reciprocating motion on the cartilage counterface, and all experiments were conducted at 37°C. A constant-load scratch test was utilized. Based on preliminary results, in order to assess the influence of normal load (F_n_) on the coefficient of friction (COF), F_n_ was varied from 5 N to 15 N, with a sliding velocity (v) of 5 mm/s. To examine the effect of sliding velocity on COF, v was set in the range of 2 mm/s to 10 mm/s, while maintaining F_n_ at 10 N. The length of each nanoindentation was 240 μm.

**Fig 1:**
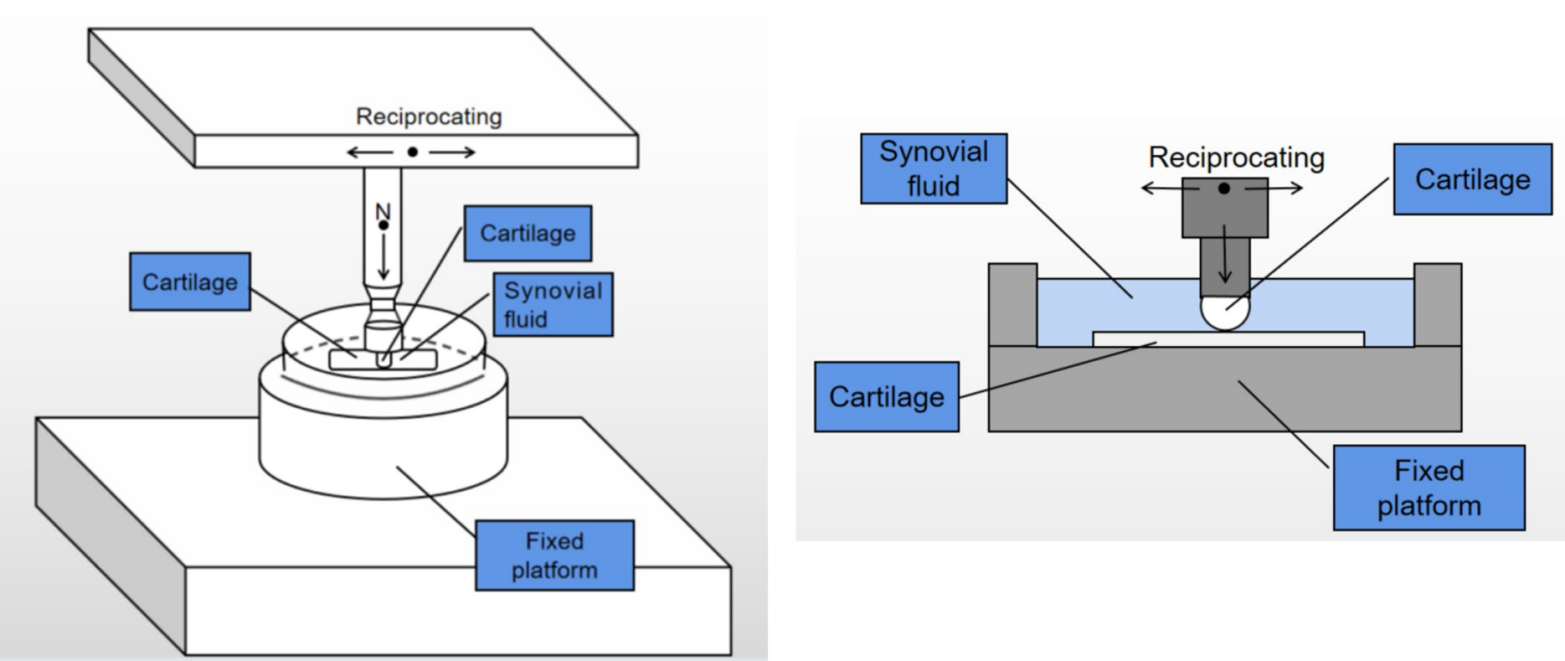
(a) Constant-load Scratch Model Diagram. (b) Model Longitudinal Cross-sectional Diagram.

For the extreme working condition of long-duration friction testing, the parameters of 10 N and 5 mm/s were employed. Post-experiment, optical microscopy (BX51-P, Olympus, Japan) was utilized to characterize the surface damage morphology of the specimens subjected to nanoindentation. Origin2021 software was used for data plotting, analysis, and processing. To ensure data reliability, each experimental set was repeated three times, and the most optimal data were selected. The experimental setup is illustrated in Fig 1.

## 3. Results

### 3.1 The impact of distinct lubricants on the coefficient of friction in cartilage

**Fig 2:**
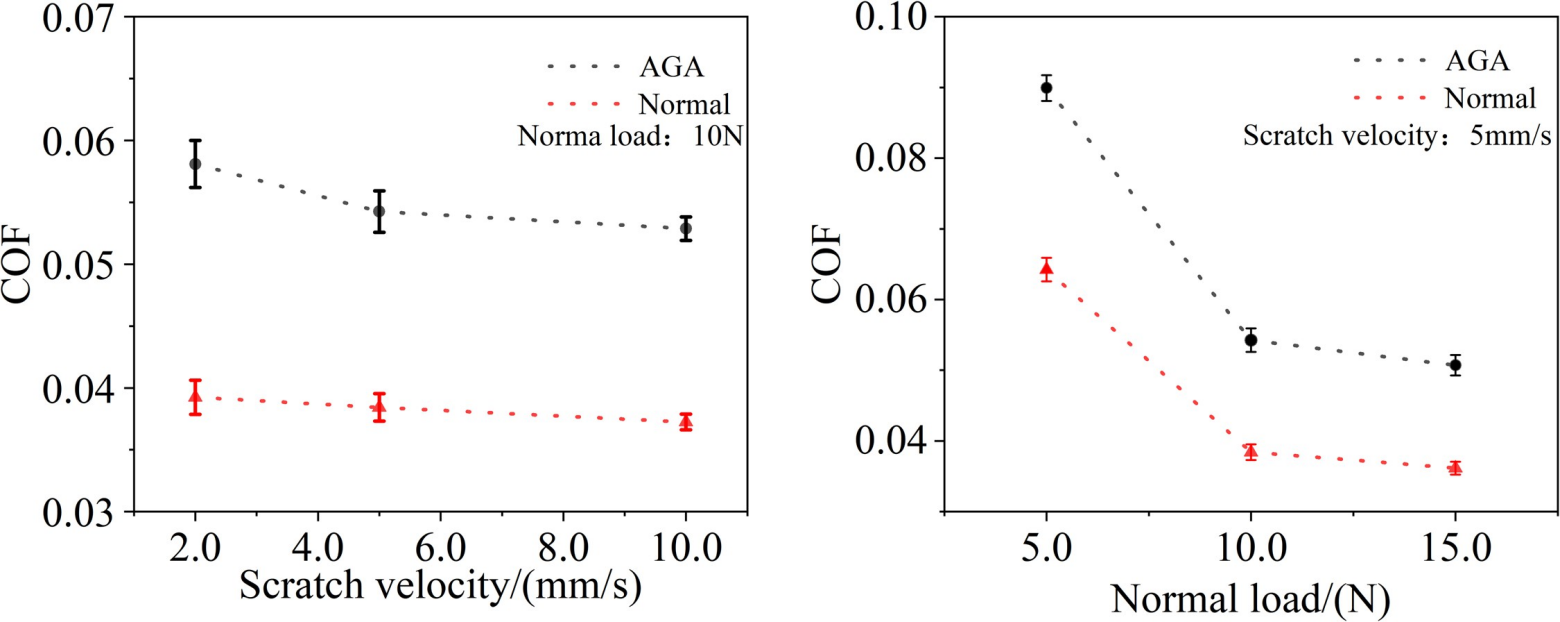
Variation of the coefficient of friction with changes in scratch velocity (a) and normal load (b).

As illustrated in Fig 2, articular cartilage is notably influenced by variations in both normal load and scratch velocity when subjected to different lubricating media, namely normal synovial fluid and synovial fluid from AGA patients. When the scratch velocity increases from 2 mm/s to 10 mm/s, the cartilage COF under lubrication by AGA patient synovial fluid decreases from approximately 0.058 to approximately 0.053, reflecting a reduction of about 9.0% (Fig 2A). Correspondingly, the COF under lubrication by normal synovial fluid decreases from about 0.039 to about 0.037, marking a decrease of approximately 5.1%.

Upon increasing the normal load from 5 N to 15 N, the friction coefficient of the AGA group drops from around 0.090 to about 0.051, presenting a substantial decline of 43.7%. Similarly, the friction coefficient of the normal group drops from approximately 0.064 to roughly 0.036, representing a decrease of 43.6% (Fig 2B). Under identical conditions of load and sliding velocity, the friction coefficient of the AGA group significantly exceeds that of the normal group. Moreover, the error margin in the AGA group under identical conditions is markedly higher than that of the normal group, suggesting a more intense friction activity in the AGA group. Additionally, as sliding velocity increases, the AGA group experiences a slightly higher decrease in friction coefficient compared to the normal group.

### 3.2 The impact of variable sliding velocities on friction coefficient

**Fig 3:**
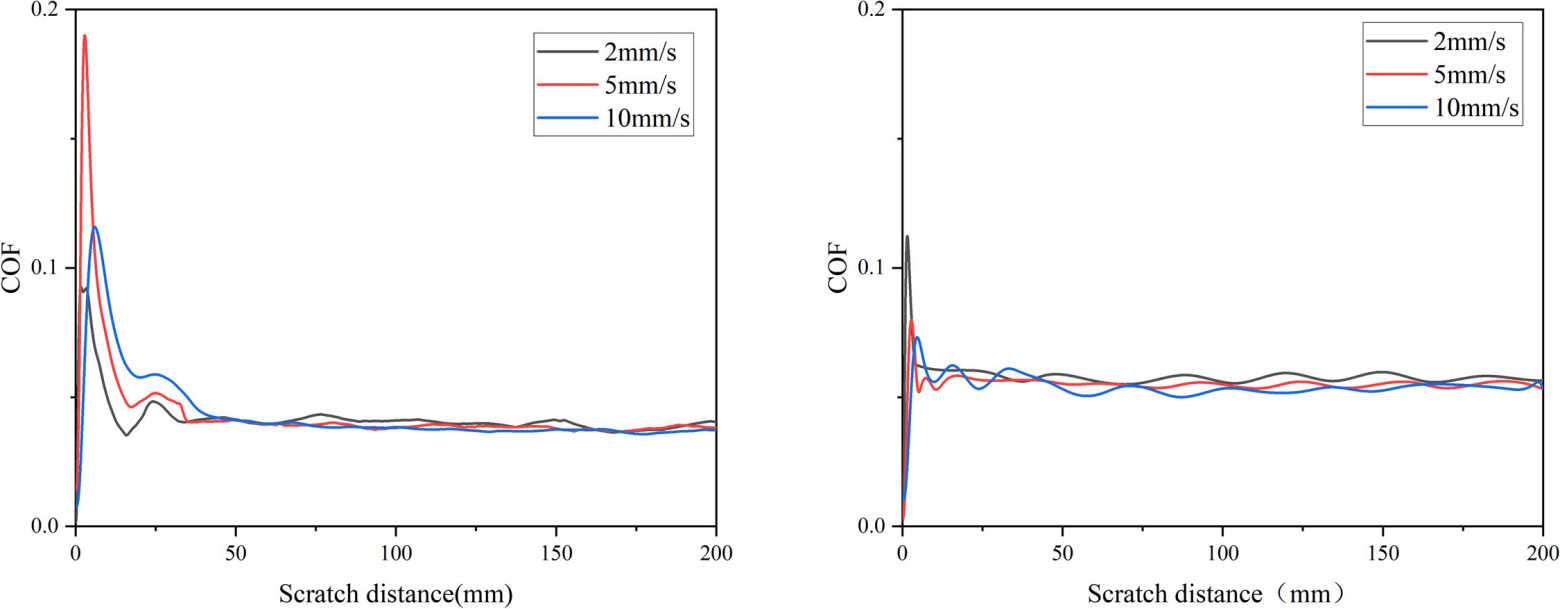
Variation of Cartilage Friction Coefficient at Different Sliding Velocities in Normal Synovial Fluid (a) and AGA Synovial Fluid (b).

As depicted in Fig 3, under a normal load of 10 N, after achieving frictional stability, at sliding velocities of 2 mm/s, 5 mm/s, and 10 mm/s, the average friction coefficients for the Normal group are approximately 0.039, 0.038, and 0.037, respectively. This represents a reduction of about 5.1% from 2 mm/s to 10 mm/s. Meanwhile, for the AGA group, the average friction coefficients are approximately 0.058, 0.054, and 0.053, respectively, showing a decrease of around 8.6%. Consequently, with an increase in scratch velocity, both groups exhibit a declining trend in cartilage friction coefficients. The higher the scratch velocity, the smoother the change in friction coefficient curves, and under the same change in sliding velocity, the AGA group experiences a slightly greater variation in friction coefficient.

### 3.3 The impact of varied normal load on friction coefficient

**Fig 4:**
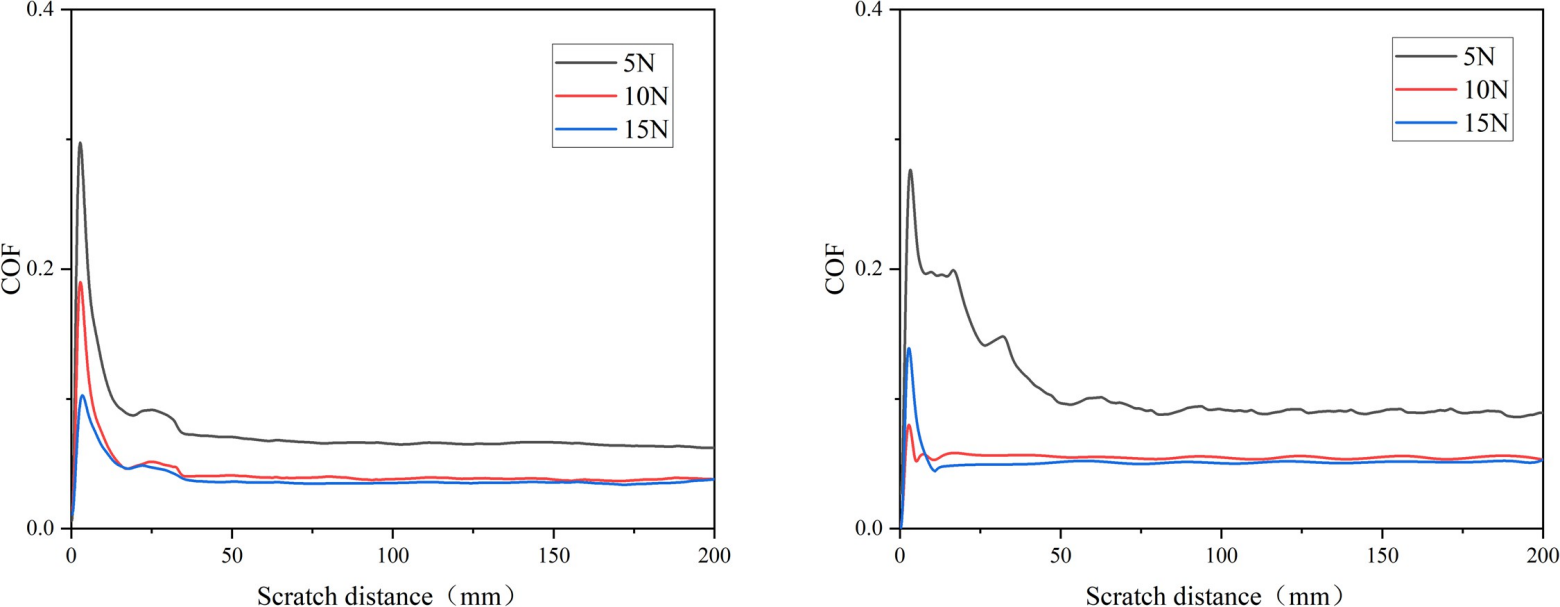
Variation of Cartilage Friction Coefficient with Different Normal Loads in Normal Synovial Fluid (a) and AGA Synovial Fluid (b).

As illustrated in Fig 4, at a scratch velocity of 5 mm/s, when the normal load is 5 N, 10 N, and 15 N respectively, the stabilized average cartilage friction coefficients in the normal synovial fluid group are approximately 0.064, 0.038, and 0.036. With an increase in the normal load from 5 N to 15 N, the reduction amounts to approximately 43.8%. In the AGA group, under the same conditions, the stabilized average friction coefficients are 0.090, 0.054, and 0.051. The reduction rate is approximately 43.3%. Therefore, with an increase in normal load, the cartilage friction coefficients decrease in both groups. Moreover, as the load increases, the variation in friction coefficient curves becomes smoother. There is no significant distinction in the extent of change in friction coefficients between the two groups under the same variation in normal load.

### 3.4 Variation of friction coefficient under extreme conditions and optical micrographs

**Fig 5:**
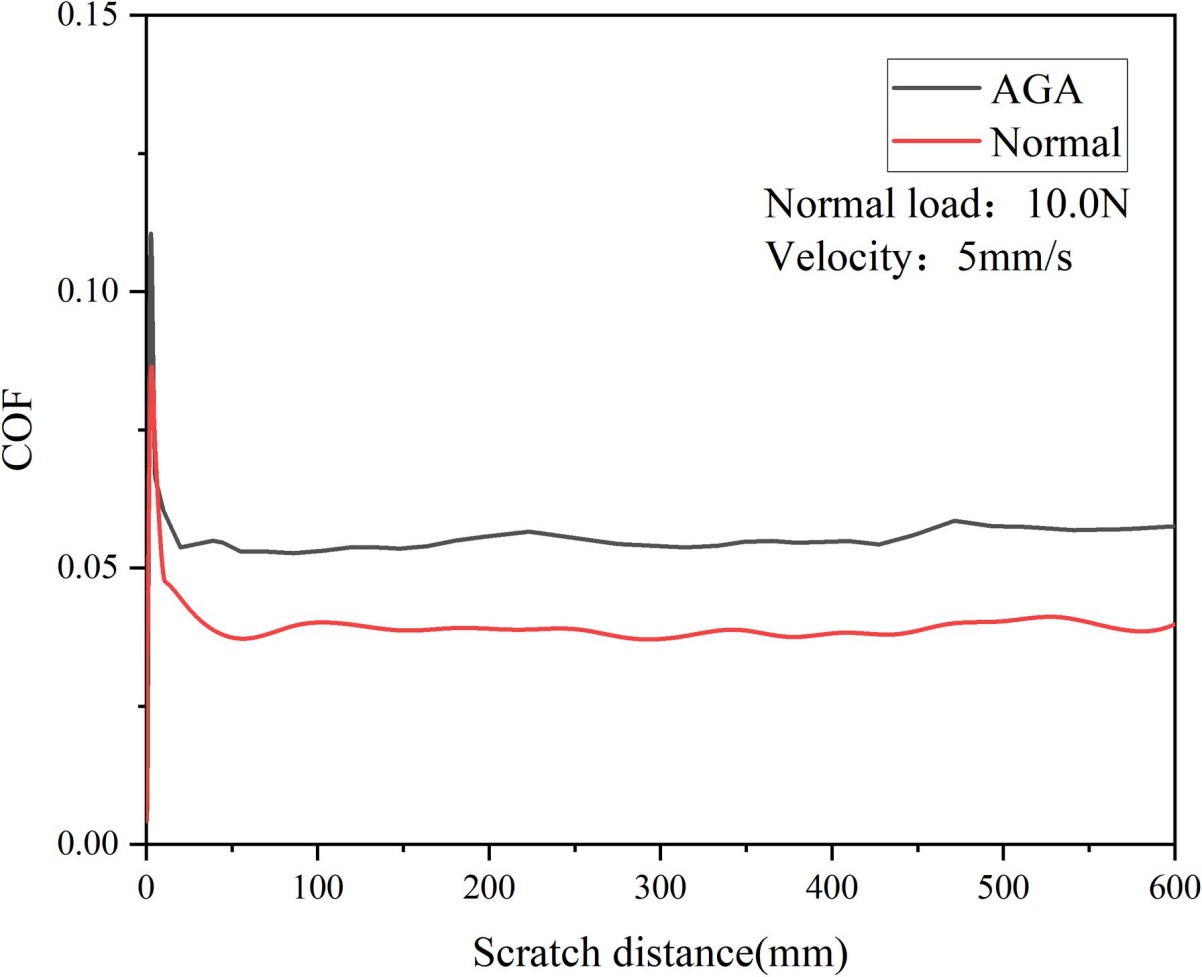
Effect of scratch distance on friction coefficient.

As demonstrated in Fig 5, slight differences arise in the variation of friction coefficients between the AGA and Normal groups for cartilage pairs under the same scratch velocity and normal load, following prolonged friction. In this scenario, the scratch velocity is 5 mm/s, and the normal load is 10 N. Upon surpassing a sliding distance of 50 mm and entering a phase of stabilized friction, the average friction coefficient for the AGA group is approximately 0.056, while for the Normal group, it’s around 0.039. Notably, the AGA group exhibits a significantly higher friction coefficient than the Normal group.

However, when the sliding distance reaches 420 mm, both groups experience a conspicuous increase in friction coefficient. Among these, the Normal group’s coefficient rises by approximately 5.3%, while the AGA group’s coefficient ascends by approximately 7.3%. Consequently, the AGA group’s friction coefficient experiences a slightly higher increment compared to the Normal group.

**Fig 6:**
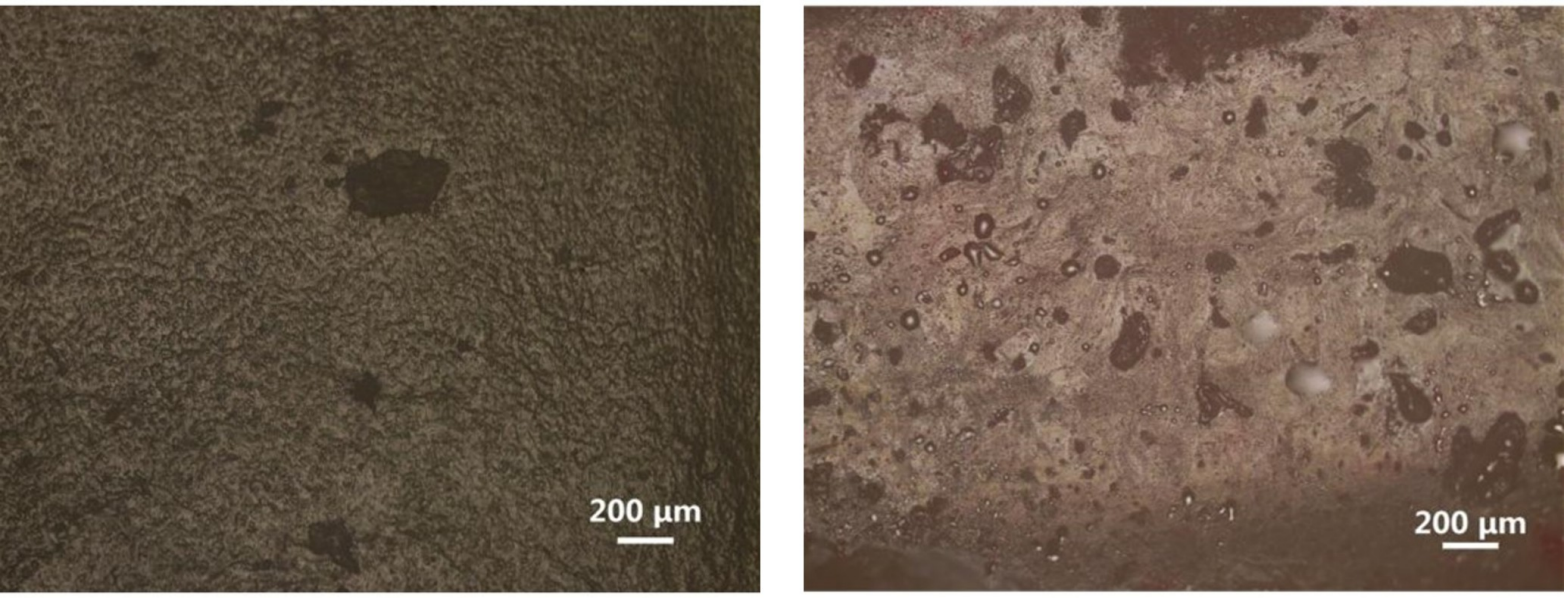
Optical Micrographs of Cartilage after Prolonged Friction under Normal Synovial Fluid (a) and AGA Synovial Fluid (b) Lubrication.

As illustrated in Fig 6, the optical micrographs of the two groups following prolonged friction exhibit considerable disparities. It is evident that although localized abrasion marks are present in the Normal group, the majority of the cartilage surfaces remain devoid of conspicuous scratches. Conversely, in the AGA group, extensive surface wear on the cartilage becomes apparent after prolonged friction.

## 4. Discussion

### 4.1 Construction of friction coefficient equation

With a smaller normal load, the cartilage surface undergoes only elastic deformation, thus the consideration of the material surface friction coefficient is limited to the adhesive friction coefficient. Under the influence of the normal load, the contact area between two surfaces is composed of the collective sum of micro-areas formed by the contact of surface profile protrusions. This configuration is referred to as the true contact area(*A*_*r*_), and due to its minuteness, it can be assumed that the pressure in the contact area of the protrusions is notably high. When the contact region experiences deformation due to substantial pressure, adhesive phenomena occur on the contact surface, giving rise to the formation of cold-welded junctions. In situations where the contact surfaces must engage in relative sliding, a tangential force is required to shear the cold-welded junctions, thereby instigating friction. The formula for calculating adhesive friction force is as follows [14]:

$$F_{f}=A_{r}\times \tau_{s}$$ (1)

**Fig 7:**
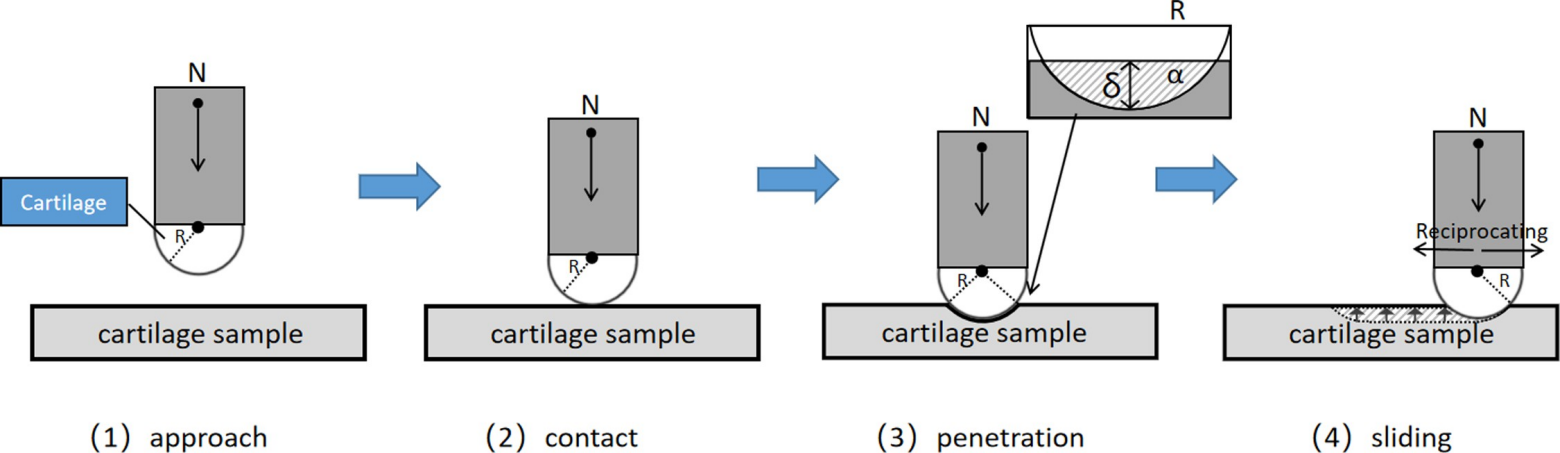
The needle tip sliding on the surface of the cartilage under elastic deformation.


Where τ_*s*_ is the ultimate shear strength of the material at the junction. Since the sliding between cartilage surfaces can be approximated as a semi-spherical surface sliding on a plane, the contact area *A*_*r*_ is influenced by the contact radius *α*, as depicted in Fig 7. By substituting *A*_*r*_ = π*α*^2^ and the radius of elastic deformation *α* = (3*F*_*n*_*R*/4*E*_*r*_)^1/3^ into formula (1) and dividing the result by the normal load *F*_*n*_, we can derive the adhesive friction coefficient *μ*:

$$\mu =\frac{F_{f}}{F_{n}}=\tau_{\text{s}}\times \pi \times {\left(\frac{3\text{R}}{4{\text{E}}_{\text{r}}}\right)}^{\frac{2}{3}}\times F_{n}^{-\frac{1}{3}}$$ (2)

Where R represents the radius of curvature of the cartilage pin, and *E*_*r*_ denotes the equivalent modulus during the sliding process. Since the cartilage used in the experiment experiences elastic deformation, the equivalent modulus is introduced to characterize the overall elastic behavior between the cartilage pin and the cartilage plane during the experimental process. The formula is as follows:

$$\frac{1}{E_{r}}=\frac{1-{\text{v}}_{s}^{2}}{E_{s}}+\frac{1-{\text{v}}_{i}^{2}}{E_{i}}$$ (3)

where *E*_*s*_, v_s_ and *E*_*i*_, v_i_ denote the modulus of elasticity and Poisson’s ratio of the cartilage tip and cartilage plane, respectively, and the tip and plane compositions are the same, so according to Eq (3), the folding modulus *E*_*r*_ can be expressed as:

$$E_{r}={\left[2\left(1-v_{\text{i}}^{2}\right)/E_{0}\right]}^{-1}$$ (4)

Where *E*_0_ and v_i_ respectively represent the elastic modulus and Poisson’s ratio of the cartilage. When the constructed cartilage friction model is subjected to compression, the cartilage in the pin region will experience pressure and permeate into the underlying cartilage specimen. At this point, sliding begins until equilibrium is reached. Hertz introduced the elastic quantity *E*_0_ [11], where the subscript 0 denotes equilibrium stability.


$$E_{0}=\frac{3\times F_{n0}\times {\text{R}}^{-0.5}\times \delta_{s}^{-1.5}}{4}$$ (5)

Where *F*_*n*_ represents the normal load, and *δ*_*s*_ denotes the penetration depth. By substituting formula (5) into formulas (4) and (2), the friction coefficient *μ* can be obtained:

$$\mu =\frac{F_{f}}{F_{n}}=\tau_{\text{s}}\times \pi \times {\left(\frac{3\text{R}}{4E_{r}}\right)}^{\frac{2}{3}}\times F_{n}^{-\frac{1}{3}}=\frac{\tau_{\text{s}}\times 2\pi \left(1-v_{\text{i}}^{2}\right)\times \text{R}\times \delta_{s}}{F_{n}}$$ (6)

Therefore, in this motion model, within a certain range, cartilage friction coefficient is positively correlated with ultimate shear strength, penetration depth, sliding speed and contact radius, and negatively correlated with normal load.

### 4.2 Impact of synovial fluid composition on AGA

The composition of synovial fluid exerts a profound influence on the pathogenesis of gouty arthritis. Normal synovial fluid is predominantly composed of abundant water, proteins, hyaluronic acid, and a minor population of cells. In contrast, synovial fluid from individuals with gouty arthritis harbors not only inflammatory cytokines but also aggregates of urate crystals, formed predominantly from MSU. Elevated concentrations of these minute, needle-like urate crystal aggregates lead to their deposition within the joint structures, including cartilage and synovial sacs. Consequently, the synovial fluid in gouty arthritis becomes turbid and interspersed with white granular particles.

Research has revealed that MSU crystals can precipitate a 30% reduction in the viability of fibroblast-like synoviocytes (FLS), pivotal cells within the synovium. Under MSU stimulation, FLS cells unleash a surge of reactive oxygen and nitrogen species, including O^2-^, H_2_O_2_, and NO, thereby perturbing the oxidative equilibrium of the microenvironment. This perturbation induces cellular damage and compromises the structural and functional integrity of the synovial lining [15]. Furthermore, the impact extends to chondrocytes, as studies have documented extensive apoptosis in cartilage cells upon MSU exposure. The underlying mechanisms might parallel the oxidative stress observed in FLS cells induced by MSU [16]. This widespread cellular stress consequently fosters local inflammation and pain.

The consequence of synovial and chondrocyte apoptosis is the pronounced impairment of joint mobility. Necrotic cells lose their biological functionality, impeding both load-bearing and lubrication roles. In the context of gouty arthritis, the infiltration of synovial fluid laden with copious MSU crystals disrupts the native lubricating environment when it reaches the articular cartilage surfaces. This perturbation acts as a foreign entity, obstructing the normal frictional interactions within the cartilage articulation. As a result, the coefficient of friction increases. This phenomenon could serve as a pivotal determinant contributing to the observed disparities in friction coefficients between the AGA and Normal groups.

### 4.3 Lubrication characteristics and influencing factors of articular cartilage

Articular cartilage, composed of highly hydrated collagen and proteoglycans, embodies a biphasic material exhibiting both solid and fluid characteristics, rendering it with high load-bearing and low friction attributes. Consequently, a dual-phase lubrication theoretical framework has been established [17]. This framework elucidates the phenomenon of reduced friction coefficients under compressive loading through mechanisms like fluid film lubrication, interstitial fluid lubrication, and boundary lubrication [18].

Research has demonstrated that interstitial fluid between chondrocytes can mitigate friction coefficients under compressive conditions [19]. Additionally, the presence of proteoglycans (PRG4) within cartilage components, highly hydrated, hydrophilicizes the hydrophobic surface of cartilage, creating an aqueous fluid film [20]. In fluid dynamic lubrication, the squeeze film effect also exerts a significant impact on biphasic lubrication [21]. During the loading process under normal force, the squeeze film effect leads to fluid pressurization. As the fluid load support ratio escalates, the friction coefficient at the onset of motion decreases. Hence, under elevated loads, cartilage exhibits diminished friction coefficients.

Bonnevie et al.’s research unveiled that material softness increases with decreasing scratch velocity [11]. Consequently, cartilage materials will experience greater deformation and augmented contact area. Conversely, overquick scratch velocities lead to increased material hardness and reduced deformation, resulting in a decline in penetration depth (δs). According to Eq (6), when the impact of a decrease in penetration depth is greater than an increase in scratch velocities, the friction coefficient will decrease. Consequently, at high scratch velocities, cartilage exhibits lower friction coefficients.

After prolonged friction, the friction coefficient of cartilage exhibits a slight increase. However, compared to common materials like crystals and metals, the variation in friction coefficient within the cartilage friction pair is relatively small. This phenomenon could be attributed to the inherent material properties of cartilage. Its high-water content contributes to significant tensile deformation and high tensile strength. When the spherical cartilage surface of the femoral condyle comes into contact with the tibial cartilage surface, it more closely resembles a face-to-face friction scenario [22], and local pressures disperse across the entire cartilage surface. Even if some wear debris is generated, the high lubricity and low friction properties of the cartilage surface prevent a substantial accumulation of wear debris within the wear tracks. Consequently, wear debris is unable to significantly damage the friction surface or create noticeable scratches. Additionally, free lipids in the joint fluid can simultaneously repair phosphatidylcholine layers on the cartilage surface that may be compromised due to friction-induced defects [23]. Despite the minor change in the friction coefficient, the alteration is more evident in the AGA group compared to the Normal group, and surface wear is more pronounced. This discrepancy could be attributed to the extensive apoptosis of cartilage and synovial cells induced by urate crystal deposition, thereby accelerating wear debris generation.

### 4.4 Impact of AGA on exercise

The low-friction properties of cartilage are not solely determined by the nature of the synovial fluid itself, but also influenced by various factors such as its composition, scratch velocity, pressure, direction of motion, temperature, and time [24]. However, in a comprehensive perspective, the friction properties of knee joint cartilage should be maintained at a low and stable level. Excessive variations in the friction coefficient can lead to instability in cartilage friction behavior [25–27]. Experimental observations have shown that the friction coefficient in the AGA group is more sensitive to changes in scratch velocity, exhibiting larger variations compared to pressure changes. Consequently, for patients with gouty arthritis, engaging in activities that involve sudden changes in speed, such as rapid running or sudden acceleration, should be minimized to promote the stability of cartilage friction coefficients.

Despite simplifying the study of friction to encompass contact, pressure, and sliding behaviors, this experiment effectively validated the proposed cartilage friction lubrication mechanisms put forth by scholars. Furthermore, a comparative analysis of the influences of gouty arthritis synovial fluid and normal joint fluid on cartilage friction coefficients was conducted. Even uric acid crystals related to gout have not yet formed a visible solid in the joints, the presence of monosodium urate crystals in the synovial fluid of gout patients can still exert a micro-level influence on friction. This influence impedes normal joint movement and exacerbates cartilage wear and tear. Consequently, for individuals afflicted with gout, while avoiding excessive activity, blood uric acid levels should be actively controlled to minimize cartilage friction and avoid premature occurrence of osteoarthritis.

Constrained by the scope of the experiment, this study solely focused on contrasting the differences between synovial fluids from patients with AGA and those from individuals with normal joints. However, it did not delve into the categorization of uric acid levels within the synovial fluid. Early-stage gout is often characterized by hyperuricemia, with blood uric acid concentration measurement serving as the most convenient routine screening method. Subsequent investigations could investigate the impact of synovial fluids from patients across various blood uric acid gradients on cartilage friction, thus examining the optimal uric acid levels for lubricating cartilage friction from a microscopic perspective. This could eventually guide clinical strategies for managing uric acid levels.

## 5. Conclusions

This study investigates nanoscale friction and wear of cartilage in different fluid environments through nanoscratch tests employing the ramping load mode. The findings reveal that, when subjected to identical loads or sliding velocities, individuals with AGA display notably elevated friction coefficients in knee joint cartilage as compared to their healthy counterparts, signifying heightened frictional engagement. The observed trend indicates a reduction in cartilage friction coefficients with escalating load and scratch velocity. In the realm of AGA, the cartilage friction coefficient exhibits heightened responsiveness to variations in scratch velocity. Upon prolonged frictional exposure, AGA patients experience a more prominent increase in cartilage friction coefficient compared to normal subjects, accompanied by heightened surface wear. Therefore, individuals afflicted with AGA should curtail activities involving sudden speed alterations, such as sprinting or abrupt accelerations, to preserve the stability of cartilage surface friction coefficients. Furthermore, active management of blood uric acid levels holds paramount importance in disease control.
